# Evaluation of the Ion AmpliSeq SARS-CoV-2 Research Panel by Massive Parallel Sequencing

**DOI:** 10.3390/genes11080929

**Published:** 2020-08-12

**Authors:** Federica Alessandrini, Sara Caucci, Valerio Onofri, Filomena Melchionda, Adriano Tagliabracci, Patrizia Bagnarelli, Laura Di Sante, Chiara Turchi, Stefano Menzo

**Affiliations:** 1 Legal Medicine Unit, Department of Biomedical Sciences and Public Health, Polytechnic University of Marche, Torrette, 60126 Ancona, Italy; f.alessandrini@univpm.it (F.A.); f.melchionda@pm.univpm.it (F.M.); a.tagliabracci@univpm.it (A.T.); 2 Virology Unit, Department of Biomedical Sciences and Public Health, Polytechnic University of Marche, Ancona, Torrette, 60126 Ancona, Italy; s.caucci@pm.univpm.it (S.C.); p.bagnarelli@staff.univpm.it (P.B.); l.disante@staff.univpm.it (L.D.S.); s.menzo@univpm.it (S.M.); 3 Legal Medicine Unit, AOU Ospedali Riuniti, Torrette, 60126 Ancona, Italy; valerio.onofri@ospedaliriuniti.marche.it

**Keywords:** SARS-CoV-2, viral genome, massively parallel sequencing, nasopharyngeal swab, COVID-19

## Abstract

Deep knowledge of the genetic features of SARS-CoV-2 is essential to track the ongoing pandemic through different geographical areas and to design and develop early diagnostic procedures, therapeutic strategies, public health interventions, and vaccines. We describe protocols and first results of the Ion AmpliSeq™ SARS-CoV-2 Research Panel by a massively parallel sequencing (MPS) assay. The panel allows for targeted sequencing by overlapping amplicons, thereby providing specific, accurate, and high throughput analysis. A modified reverse transcription reaction, which consists of the use of a SARS-CoV-2 specific primers pool from the Ion AmpliSeq SARS-CoV-2 Research Panel, was assessed in order to promote viral RNA specific reverse transcription. The aim of this study was to evaluate the effectiveness of the Ion AmpliSeq™ SARS-CoV-2 Research Panel in sequencing the entire viral genome in different samples. SARS-CoV-2 sequence data were obtained from ten viral isolates and one nasopharyngeal swab from different patients. The ten isolate samples amplified with 12 PCR cycles displayed high mean depth values compared to those of the two isolates amplified with 20 PCR cycles. High mean depth values were also obtained for the nasopharyngeal swab processed by use of a target-specific reverse transcription. The relative depth of coverage (rDoC) analysis showed that when 12 PCR cycles were used, all target regions were amplified with high sequencing coverage, while in libraries amplified at 20 cycles, a poor uniformity of amplification, with absent or low coverage of many target regions, was observed. Our results show that the Ion AmpliSeq SARS-CoV-2 Research Panel can achieve rapid and high throughput SARS-CoV-2 whole genome sequencing from 10 ng of DNA-free viral RNA from isolates and from 1 ng of DNA-free viral RNA from a nasopharyngeal swab using 12 PCR cycles for library amplification. The modified RT-PCR protocol yielded superior results on the nasopharyngeal swab compared to the reverse transcription reaction set up according to the manufacturer’s instructions.

## 1. Introduction

SARS-CoV-2, first isolated in Wuhan, China, in December 2019 [1] is a new coronavirus responsible for the severe pulmonary/systemic disease called COVID-19. Since December 2019, the virus rapidly spread in Southeast Asia and subsequently in Europe and the rest of the world. In Italy, the first two cases were Chinese tourists diagnosed on 29 January 2020 [2], while the first documented case of local transmission, in Lombardy, was on 22 February 2020. In the same week, several clusters were discovered in northern regions (Lombardy, Emilia-Romagna, and Veneto), followed by rapid spread of the epidemic towards central regions such as Marche. Here, the first COVID-19 patient was diagnosed by the Virology Laboratory of Ancona University Hospital on the 25 February 2020.

Following the spread of the infection in Europe and all over the globe, the World Health Organization [3] declared a state of pandemic on 11 March 2020. By 29 April 2020, the reported, confirmed cases worldwide reached 3.1 M, 6.5% of which were from Italy, whereas the proportion of Italian deaths was higher, making up 13% of the 208,112 deaths worldwide, causing a deep impact to public health and the socio-economic systems. Since the first diagnosis, several viral isolates have been obtained in our laboratory from different patients and different specimens of the same patient. Whole genome sequencing, starting from viral isolates and original specimens, was immediately performed, considering that early diagnosis, therapies, and vaccine development depend on the deep knowledge of the genetic characteristics of the virus.

Furthermore, sequencing is necessary to track the ongoing pandemic through the different geographical areas. Several research centres started sequencing programs on different platforms and some results are already available [2,4,5,6] in databases such as Global Initiative on Sharing All Influenza Data (GISAID, https://www.gisaid.org/) or GenBank (https://www.ncbi.nlm.nih.gov/genbank/sars-cov-2-seqs/), which will continue to accumulate sequencing data from around the world.

Massively parallel sequencing (MPS) provides both fast whole genome sequencing and, thanks to sequencing redundancy, fine quasispecies analysis allowing for the identification and quantification of both major and minor variants.

In this study, we describe a protocol and the first results of an MPS assay based on Ion AmpliSeq™ technology on the Ion Torrent NGS platform (Thermo Fisher Scientific, Waltham, MA, USA). The Ion AmpliSeq™ SARS-CoV-2 Research Panel allows for targeted sequencing by overlapping amplicons of the entire viral genome. Very few studies concerning this panel have been published until now [7,8,9,10], therefore, we decided to investigate its effectiveness through sequencing of viral genomes obtained from ten viral isolates and one nasopharyngeal swab from different patients.

For the nasopharyngeal swab, we applied a modified reverse transcription reaction, where a SARS-CoV-2 specific primers pool from the Ion AmpliSeq SARS-CoV-2 Research Panel (Thermo Fisher Scientific) was used in order to promote viral RNA specific reverse transcription, rather than using a universal primer set as suggested in the Ion AmpliSeq Library Kit 2.0 user guide.

This novel method allows for targeted sequencing of amplified products, thereby providing specific, rapid, and high throughput analysis. This method proved very effective for sequencing the whole SARS-CoV-2 genome from both cell cultures and a nasopharyngeal swab.

## 2. Materials and Methods

### 2.1. Ethical Approval

According to the ethical directives of the Marche Polytechnic University, an opinion from the ethics committee was not necessary as this is a technological study for diagnostic procedures performed on samples obtained for homogeneous purposes (molecular diagnostics of SARS-CoV-2), moreover, the samples were detached from a clinical context and anonymized. Each patient’s informed consent to collection and storage of surplus samples for scientific purposes was obtained.

### 2.2. SARS-CoV-2 Isolation

Nasopharyngeal swabs collected at Ospedali Riuniti of Ancona (Italy) from 10 patients (6 females and 4 males; median age 57, range 32–81) who presented to the medical guard/hospital ER with respiratory symptoms from 26 February to 18 March 2020 and were tested positive by RT- PCR test assay for SARS-CoV-2 were selected for the present study (sample IDs: #32898, #34104, #34117, #43950, #71205, #73702, #78952, #73696, #77488, #78955).

Isolation of SARS-CoV-2 was performed using Vero E6 cells as follows: a swab aliquot (0.5 mL) was mixed with 2 × 10^6^ Vero E6 cell suspension in 2 mL of complete medium. After incubation for 1 h at 37 °C, 5% CO_2_, 4 mL of complete medium was added, and the suspension was transferred into a 25 cm^2^ tissue culture flask (CELLSTAR, Greiner Bio-One, Frickenhausen, Germany). Virus-infected cells were maintained at 37 °C in 5% CO_2_ in DMEM (DMEM, Euroclone, Milano, Italy) supplemented with 10% FCS (FCS, Euroclone) and antibiotic/antimycotic (Euroclone). Three days after inoculation, the cell cultures were examined with an optical microscope (magnification 10× or 20×) to identify the typical cytopathic effects (CPE), consisting of rounding and detachment of cells.

### 2.3. RNA Extraction

Vero E6 cells, seeded in 75 cm^2^ flasks, were inoculated with 2 mL of the virus from the isolation vessel to a final volume of 12 mL in order to obtain a larger stock. Three days after infection, the infected cell suspensions (12 mL) were centrifuged at 3000 rpm for 10 min to remove cellular debris; supernatants were further ultra-centrifuged at 18,000 rpm for 45 min in order to obtain pellets with high viral concentration.

Viral RNA was extracted from nasopharyngeal swab (#78955-sw) or pellet (#32898, #34104, #34117, #43950, #71205, #73702, #78952, #73696, #77488, #78955) using the Kit QIAsymphony DSP Virus/Pathogen Midi kit on the QIAsymphony automated platform (QIAGEN, Hilden, Germany) according to manufacturer’s instructions and stored at −80°C until use. For the sake of simplicity, in this paper, the viral RNA extracted from cell cultures is named the “isolate”.

### 2.4. Quantification of SARS-CoV-2 by Real-Time PCR

The extracted RNA was reverse transcribed to cDNA and subsequently amplified on the Applied Biosystems™ 7500 Fast Dx Real-Time PCR Instrument (Thermo Fisher Scientific) applying a protocol described by the CDC (https://www.fda.gov/media/134922/download). A calibration curve, obtained from 10-fold dilutions (10^5^ to 10^3^ copies per reaction) of a standard plasmid certified and quantified by a supplier (2019-nCoV Positive Control, nCoVPC, IDT), was included in each real-time session along with a negative control. As expected, isolate samples showed a very high viral RNA concentration, as did the nasopharyngeal swab (viral titre: 1.37 × 10^7^ copies/μL, real-time PCR Ct = 14).

### 2.5. DNase-Treated and Complementary DNA (cDNA) Synthesis

For each sample, 500 ng of viral RNA from isolates and 10 ng or 0.1 ng of viral RNA from a nasopharyngeal swab were subjected to DNase treatment using DNase I, Amplification Grade (Invitrogen, Waltham, MA, USA), according to the manufacturer’s instructions. In brief, 1 µL of 10X DNase I Reaction Buffer, 1 µL of DNase I, and DEPC-treated water up to a final volume of 10 µL were added to viral RNA. After a 15 min incubation at room temperature, 1 µl of 25 mM EDTA solution was added to the reaction mixture to inactivate the DNase I, and the samples were heated in a Applied Biosystems™ Veriti™ 96-Well Thermal Cycler (Thermo Fisher Scientific) for 10 min at 65 °C. Viral RNA from isolates was reverse transcribed using Invitrogen™ SuperScript™ VILO™ cDNA Synthesis Kit (Thermo Fisher Scientific), according to the Appendix B supplemental procedures in the Ion Torrent™ Ion AmpliSeq™ Library Kit 2.0 (Thermo Fisher Scientific) protocol (Thermo Fisher Scientific, MAN0006735 rev F.0): 2 µL of 5X VILO™ Reaction Mix, 1 µL of 10X SuperScript™ Enzyme Mix, viral RNA (10 ng), and DEPC-treated water to a final volume of 10 µL. This RT protocol is referred to as “A” in Table 1. The RT reaction was performed in a Veriti™ 96-Well Thermal Cycler (Applied Biosystems, Thermo Fisher Scientific) at 42 °C for 30 min followed by 5 min at 85 °C.

For the nasopharyngeal swab with 10 ng of viral RNA as the input, two different reverse transcription reactions were performed: one according to the Appendix B supplemental procedures in the Ion AmpliSeq Library Kit 2.0 user guide as previously described (sample #78955-sw1) with 1 µL of DNA-free viral RNA (~1 ng) as input, the other by a modified protocol (sample #78955-sw). Firstly, the reaction mixture was set up with the SARS-CoV-2 specific primers pool from the Ion AmpliSeq SARS-CoV-2 Research Panel (Thermo Fisher Scientific) and an initial step at 50°C for 5 min was introduced in order to promote viral RNA specific reverse transcription. Therefore, cDNA synthesis was performed in two different tubes, both containing 2 µL of 5X VILO™ Reaction Mix, 1 µL of 10X SuperScript™ Enzyme Mix, 1 µL of DNA-free viral RNA (~1 ng), 5 µL DEPC-treated water, and with either 1 µL of 5X primer pool 1 or 2. This RT protocol is referred to as “B” in Table 1. The RT reaction was performed in a Veriti™ 96-Well Thermal Cycler (Applied Biosystems, Thermo Fisher Scientific) at 50 °C for 5 min, 42 °C for 25 min, and a final step at 85° for 5 min. One replicate (sample #78955-sw2) of this sample was performed in order to check the reproducibility of this method. The nasopharyngeal sample with 0.1 ng of viral RNA input (#78955-sw0.1) was retrotranscribed according to the modified protocol.

### 2.6. MPS Libraries Preparation and Sequencing

Libraries for the MPS assay were prepared using the Ion AmpliSeq SARS-CoV-2 Research Panel designed by Thermo Fisher Scientific for complete viral genome sequencing. This panel is based on a targeted approach by the amplicon method and consists of 247 amplicons, of which 237 are specific to the SARS-CoV-2 sequence, with insert sizes (sequenced regions) ranging from 54 to 232 bp, and the other amplicons that map in five human regions were used as control. The panel consists of two primer pools: primer pool 1 containing 125 primer pairs (120 specific to the SARS-CoV-2 and 5 human specifics) and primer pool 2 containing 122 primer pairs (117 specific to the SARS-COV-2 and 5 human specifics). The amplicons’ lengths, ranging from 125 to 275 bp, allow for SARS-CoV-2 sequencing to be performed on Ion Torrent platforms.

Note that the panel design allows for the sequencing of 99% of the SARS-CoV-2 genome, covering from position 43 to position 29,842 (positions related to reference sequence [1]). The panel misses only the first 42 and the last 61 nucleotides of SARS-CoV-2 sequence to account for primer placement. Moreover, the panel is designed using an overlapping amplicon strategy, which allows 62.78% of the viral genome to be sequenced twice, by using different primer pairs. This strategy allows for greater accuracy in sequence variant assignment, as the presence of a variant in only one of the two amplicons that covers a specific region represents an alert for potential sequencing artefacts and highlights the need for further sequence inspections.

All information about the Ion AmpliSeq SARS-CoV-2 Research Panel are available at https://ampliseq.com.

Libraries were prepared according to the Ion AmpliSeq™ Library Kit 2.0 user guide (Thermo Fisher Scientific, MAN0006735 rev F.0), following the Ion AmpliSeq™ RNA libraries protocol. All the reactions were performed in an Applied Biosystems™ Veriti™ 96-Well Thermal Cycler (Thermo Fisher Scientific).

**Figure 1:**
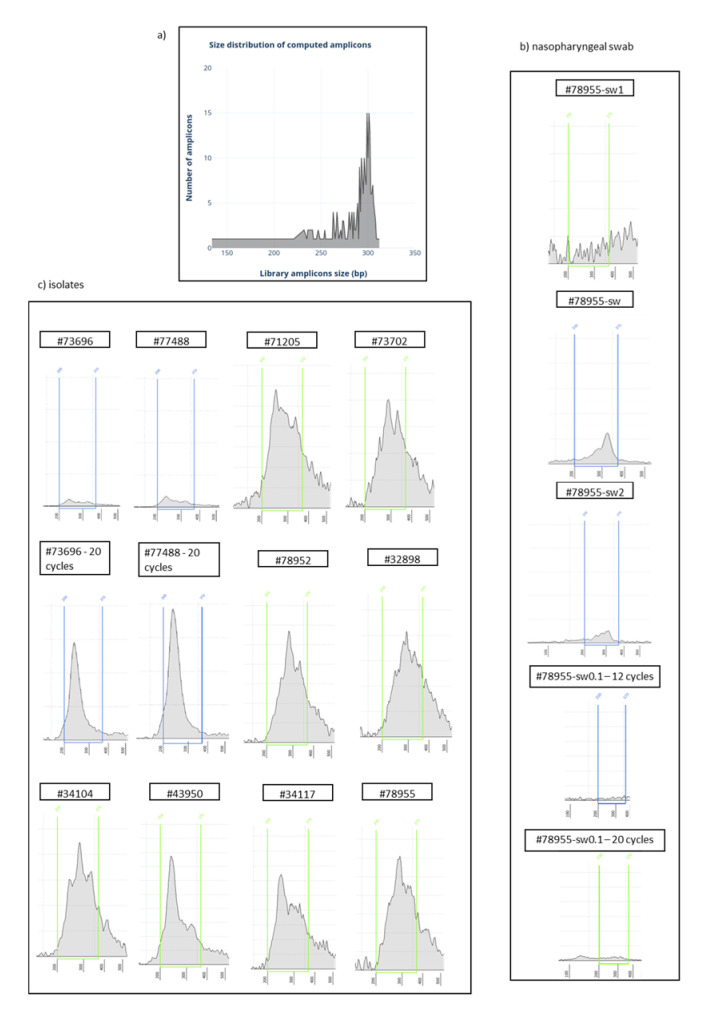
(**a**) Size distribution of computed amplicons of the Ion AmpliSeq SARS-CoV-2 Research Panel. (**b**,**c**) Size distribution of each barcoded cDNA library determined on the Agilent TapeStation 2200 using the Agilent High Sensitivity D1000 ScreenTape Assay in the nasopharyngeal swab (**b**) and isolates (**c**). The 200–370 bp size range was considered for library concentration assessment. Unless otherwise noted, samples were amplified by using 12 PCR cycles. Sample #78955-sw was reverse transcribed by using a modified protocol (see Material and Methods section for details), and it displays a profile most similar to that which was expected (**a**).

For isolates and sample #78955-sw1, the cDNA target amplification reaction was set up using 5 μL of cDNA, 4.5 μL of 5X Ion AmpliSeq™ HiFi Mix, and 8.5 μL of nuclease-free water; this mixture was then divided into two different tubes and 2  μL of each of the 5X Ion AmpliSeq™ Primer Pool 1 and 2 were added to the corresponding tubes. For the nasopharyngeal swabs #78955-sw, #78955-sw2, and #78955-sw, 0.1, 5 μL of each cDNA obtained were amplified with 2.25 μL of 5X Ion AmpliSeq™ HiFi Mix, 1 μL of the corresponding 5X Ion AmpliSeq™ Primer Pool, and 1.75 μL of nuclease-free water. Thermal cycling was performed using the following conditions: enzyme activation for 2 min at 98  °C, followed by 12 cycles at 98 °C for 15 s and 60  °C for 4 min. Samples #78955-sw0.1, #73696, and #77488 were also amplified by 20 PCR cycles. The target amplification reactions from each sample were then combined together and 2 μL FuPa Reagent were added to partially digest the primers (Thermo Fisher Scientific), and the mixture was incubated for 10 min at 50  °C, for 10 min at 55 °C, and for 20 min at 60 °C. Then, 2 μL of diluted Ion Xpress™ Barcode Adapters together with 4 μL of Switch Solution and 2 μL DNA Ligase were added to ligate the adapters to the amplified products, and the samples were incubated for 30 min at 22 °C, 5 min at 68 °C, and 5 min at 72 °C. After ligation, each library was purified with the Agencourt™ AMPure™ XP Reagent (Beckman Coulter, Brea, CA, USA) and then amplified with 50 μL of Platinum™ PCR SuperMix HiFi and 2 μL of Library Amplification Primer Mix using the following conditions: 2 min at 98 °C, 5 cycles of 15 s at 98 °C and 1 min at 64 °C. The amplified libraries were purified with Agencourt™ AMPure™ XP Reagent (Beckman Coulter), and the final concentration of each barcoded cDNA library was determined on the Agilent TapeStation 2200 using the Agilent High Sensitivity D1000 ScreenTape Assay, following the manufacturer recommendations. Ion Ampliseq libraries have an additional ~80 bp due to barcode adapters (MAN0015830 Revision B.0), therefore, to better describe the representation of the expected amplicon size of the panel, the 80 bp representing the barcodes and adapters ligated during the libraries preparations were virtually added to each insert, and the results were plotted to describe the distribution of the amplicons with identical size. Figure 1a shows the quantitative representation of the amplicons by size, with the majority of target regions being around 300 bp. This distribution was used to estimate the accuracy of the prepared libraries: the closer the observed distribution fits the expected one, the more accurate is the library. Library concentration was determined in the 200–370 bp size range and was expressed as region molarity.

Barcoded libraries were diluted to 40 pM, pooled in equal volume aliquots, and then loaded on to the Ion Chef™ Instrument (Thermo Fisher Scientific) for emulsion PCR, enrichment, and loading onto the Ion S5 520 chip.

Three sequencing runs were performed on the Ion GeneStudio™ S5 System (Thermo Fisher Scientific), the first chip contained the replicates at 12 and 20 cycles of two isolates (sample #73696 and sample #77488), the second contained 5 isolates, and the last one contained 3 isolates and the nasopharyngeal swab.

### 2.7. MPS Data Analysis

Reads were aligned with the Wuhan-Hu-1 NCBI Reference Genome (Accession number: MN908947.3) on the Torrent Suite v. 5.10.1. The following plugins were used: Coverage Analysis (v5.10.0.3), Variant Caller ( v.5.10.1.19) both with “Generic - S5/S5XL (510/520/530) - Somatic - Low Stringency” and “Generic-S5/S5XL (510/520/530)-Germ Line-Low Stringency” default parameters and COVID19AnnotateSnpEff (v.1.0.0), a plugin specifically developed for Sars-Cov-2 that can predict the effect of a base substitution. The software Integrative Genomic Viewer_2.8.0 (IGV) [11] was used to visualize the TVC (torrent variant caller) bam files of each sample in order to check the consistency of nucleotides calls.

## 3. Results and Discussion

### 3.1. Libraries Assessment

All libraries prepared from isolates and three libraries prepared from a nasopharyngeal swab (#78955-sw) displayed positive results at the quantification assay, with high region molarity (Table 1), despite differences in the relative distribution of the amplified products as revealed by relative electrophoretic patterns. At first, we evaluated the samples #73696 and #77488 amplified with 12 and 20 PCR cycles, to determine the suitable number of PCR cycles. The two isolates amplified with 20 PCR cycles showed higher concentration (6–7 times) compared to replicates of the same samples amplified with 12 PCR cycles. Despite the higher concentrations, the shapes of electrophoretic curve revealed a poor uniformity of target regions amplification after 20 cycles, with an overrepresentation of regions around 240 bp, while libraries prepared at 12 cycles showed a wider curve, covering amplicons of all the expected sizes (Figure 1c). All the remaining libraries amplified with 12 PCR cycles displayed curves ranging between 200 and 370 bp and high molarity. Interestingly, the library prepared from the nasopharyngeal swab, reverse transcribed with a modified protocol by using the SARS-CoV-2 specific primer pools from the Ion AmpliSeq SARS-CoV-2 Research Panel, displayed the best amplicon curve distribution and was very similar to the expected pattern (Figure 1b). Moreover, as further detailed in the next sections, a correlation between accuracy in amplicon distributions and sequencing uniformity of viral genome was observed.

The comparison between the curve of the nasopharyngeal swab (sample #78955-sw) and its replicate with the same reaction conditions (sample #78955-sw2) did not show a difference in the distribution of the amplicons (Figure 1b); this demonstrated that the method was reproducible. This sample was not sequenced. The nasopharyngeal swab (#78955-sw0.1) amplified with 12 PCR cycles displayed negative results at the quantification assay (the final concentration of 71.2 pM was originated by the background noise of the electrophoretic assay), while at 20 PCR cycles it showed a wider curve, ranging between 120 and 370 bp, more similar to the expected pattern, but the longest amplicons were still underrepresented (Figure 1b). Despite this sample not being sequenced, the quantification results would suggest that better results could be obtained by using 20 PCR cycles with low viral titre samples.

Finally, the library prepared from the nasopharyngeal swab and reverse transcribed according to the Appendix B supplemental procedures in the Ion AmpliSeq Library Kit 2.0 protocol (MAN0006735 rev F.0) did not yield any results, and the final concentration of 657 pM was originated by the background noise of the electrophoretic assay (sample #78955-sw1 in Figure 1). As a result, this library was discarded.

In order to confirm and strengthen the genome sequences generated, the sample #77488 was also submitted to whole transcriptome RNA sequencing (data not shown) with Ion Total RNA-Seq Kit v2 (Thermo Fisher Scientific). The results between the two sequencing methods were compared, and identical sequences were obtained, with the same nucleotide variants observed.

### 3.2. Ion Gene Studio S5 Sequencing Summary

A total of 13 libraries were sequenced on three 520 ion chips: two chips were loaded with 4 libraries each and one with 5 libraries.

A summary of the runs and alignments of the three sequencing sessions is shown in Table 2. Ion Sphere Particles (ISP) loading was 93.77% on average and 98.63% of the ISP was represented by libraries. About 28.47% of the ISPs was polyclonal, while low quality products and adapter dimers represented 5.17% and 0.03% of the sequences, respectively. On average, the final libraries’ ISPs were 66.37% of the total. Aligned reads against the SARS-Cov-2 reference sequence represented almost the total of the reads, with a very low amount of reads not aligned to the reference genome. These unaligned reads are not specific sequencing products and probably arose from primers dimerization during the PCR amplification step. However, due to their low incidence, these unaligned reads were not further investigated and characterized.

Overall, these results show the optimal quality of the sequenced libraries, with all live ISPs and a high percentage of final libraries’ ISPs demonstrating the high performance of the panel in terms of target amplification and sequencing specificity.

### 3.3. Mean Depth and Locus Coverage Balance

The ten isolates samples amplified by 12 PCR cycles displayed high mean depth values, with values ranging from 5727 to 34,209, compared to those of the two isolates amplified by 20 PCR cycles, which displayed mean depth values equal to 57.1 and 108.9 (Table 1). Moreover, considering the direct comparison of 12 and 20 cycles of the two isolates (sample #73696 and sample #77488), great differences in mean depth were observed (34,209 vs. 108.9 for sample #73696 and 12,087 vs. 57.1 for sample #77488, respectively). High mean depth values were also obtained for the nasopharyngeal swab (#78955-sw).

**Figure 2:**
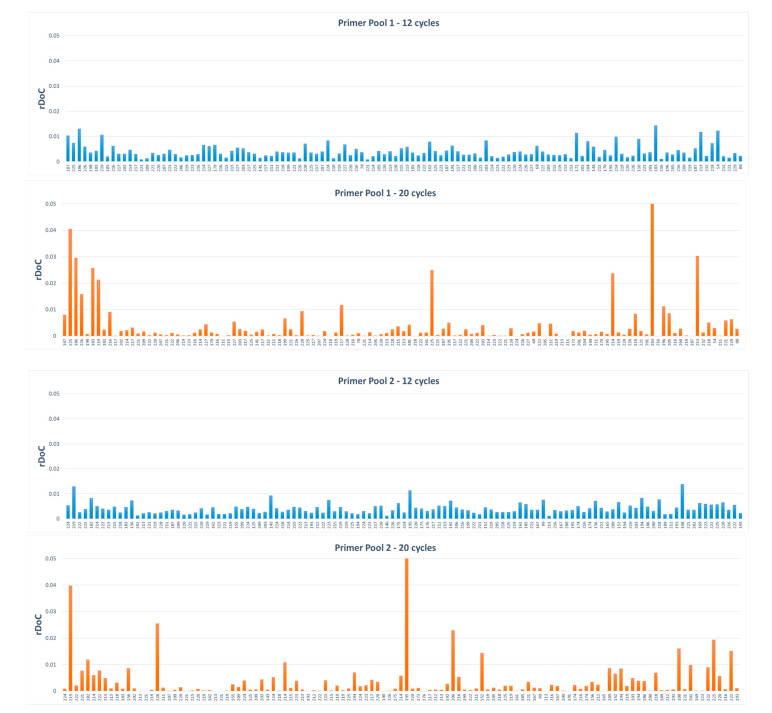
Mean relative depth of coverage (rDoC) distribution observed in libraries from isolates amplified with 12 and 20 PCR cycles. For easier viewing, the distributions are separated into two plots relative to the different primer pools.

The locus balance among all amplified regions was determined in order to check the amplification efficiency at each locus, comparing libraries amplified with different numbers of PCR cycles and also comparing the results obtained by analysing the viral genome isolated from cell cultures and the one directly purified from the nasopharyngeal swab from the same patient. The parameter considered for this purpose was the relative depth of coverage (rDoC), which is the ratio between the coverage of each locus versus the overall coverage of the sample. Figure 2 shows the distribution of the mean rDoC observed for primer pool 1 and primer pool 2, amplified with 12 or 20 PCR cycles. Libraries amplified at 20 cycles display a very poor uniformity of amplification, with many target regions not amplified or with low coverage, both considering primer pool 1 and primer pool 2. When 12 PCR cycles were used, all target regions were amplified, and high sequencing coverage was observed for both primer pools, even if rDoC values were not uniform across all regions (Figure 2). Despite the lack of uniformity at certain loci, high mean depth values were observed in all regions, with a minimum coverage of 771x observed in one region in one isolate sample. By contrast, the low mean depth and the great unbalance in target region amplification observed after 20 PCR cycles could be explained by preferential amplification at certain viral genome regions and production of chimeric reads due to associated primer depletion, which results in a poor performance of the Ion AmpliSeq SARS-CoV-2 Research Panel using 5 µl of cDNA obtained from 10 ng of DNA-free viral RNA. Although the observation was limited to only two samples, apparently in these two samples the regions that were poorly amplified coincided, without any correlation with amplicon size or specific viral genome sites.

Samples #34104 and #78955 displayed a very low coverage (67× and 74×, respectively) at region r1_1.5.1289446, despite a high mean depth (15651 and 7757, respectively). In region r1_1.5.1289446 (positions 4019-4239 on the viral genome) both samples displayed a G4255T substitution in 100% of the reads. The position 4255 is 15 nucleotides away from the end of the region, therefore, it likely maps to one of the primer annealing sites (the exact primer sequence of the panel is unknown). As a result, the low PCR efficiency of the r1_1.5.1289446 target region in these two samples could be explained by a nucleotide substitution in the primer annealing region, which results in a decrease of annealing efficiency. Moreover, a library from sample #78955 was also prepared by using a modified protocol for reverse transcription (#78955-sw), and surprisingly, in this library, the r1_1.5.1289446 was regularly amplified, despite the presence of the substitution in 100% of the reads. This observation might be due to the specific reverse transcription step of the library (#78955-sw), which may have enhanced the proportion of sequences without the 4255 variant (arose from primer elongation during the RT process), resulting in a better annealing of the same primer during the PCR amplification of the r1_1.5.1289446 target.

The Ion AmpliSeq SARS-CoV-2 Research Panel was designed to amplify 237 target regions and, therefore, there are 474 different primer sites mapping to the SARS-CoV-2 genome. At the time of panel design, poor information about sequence variations originated and accumulated during the early spread of the virus and, therefore, nucleotide substitutions occurring at primer sites cannot be excluded. Although the algorithm used for the design of the primers took into account the eventuality of single sequence mismatches in the primer regions, which should not compromise correct annealing, a decrease in amplification efficiency at these regions can be observed. Therefore, we performed a deep and accurate coverage analysis at each amplified target in order to identify any poor sequenced regions, which could affect the correct genotyping.

Viral variants calling was performed by applying a threshold of 10% with respect to the total position coverage as a minimum frequency of the minor variants. As a result, we detected eleven minor viral variants in six different samples. The genetic characterization of the viral variants found in this study was not the subject of this report and was published in Lai et al. [12].

It is difficult to draw a comparison between alternative methods since only a few multiplex amplicon-based papers to sequence the whole SARS-CoV-2 genome have been published [13,14]; in these studies, the panels were in the 150–400 bp amplicon range and designed to be used with MGI or Illumina platforms. When compared to a hybrid capture-based method, the authors in [13] observed low uniformity of coverage across different samples, although the amplicons method was more efficient than capture-based one for more challenging samples. Finally, one study [10] analysed five clinical samples by a similar approach as that used in the present study, and results showed closely similar mapped reads and depth of coverage values.

## 4. Conclusions

In this study, we describe the protocols and the first results of a targeted MPS assay by Ion GeneStudio™ S5 System. The Ion AmpliSeq SARS-CoV-2 Research Panel allows for targeted sequencing by amplicons, providing specific, accurate, and high throughput analysis of the SARS-CoV-2 whole viral genome. Our results show that optimal cDNA libraries can be obtained after reverse transcription of 10 ng of DNA-free viral RNA purified from supernatants of viral isolates in tissue culture. The standard reverse transcription method (Appendix B supplemental procedures in the Ion AmpliSeq Library Kit 2.0 user guide), based on random primers, apparently works best on concentrated samples, while it performed poorly on a clinical sample that was less concentrated than viral isolates. Therefore, a modified RT-PCR protocol was developed for enhancing the reverse transcription step based on specific primers, as described in detail. By this procedure, optimal cDNA libraries can be generated from 1 ng of DNA-free viral RNA, which allows for direct sequencing from clinical samples with lower viral loads, such as nasopharyngeal swabs.

For all cDNA libraries, the best amplification results, in terms of amplicon representation and uniformity of depth, were achieved after 12 amplification cycles, while at 20 cycles, some bias in the differential amplification efficiency within the pool of primer pairs caused some skewing of the amplified products. The results also highlight the need for a close scrutiny of the coverage at each sequenced target region. Any isolate-specific bias in the amplification efficiency of the panel, independently of the number of amplification cycles, could be due to mutations in the primer annealing regions.

In conclusion, the Ion AmpliSeq SARS-CoV-2 Research Panel is a useful and versatile tool for whole genome MPS of the SARS-CoV-2 genome, both from isolates and from nasopharyngeal swabs.

## Figures and Tables

**Table 1:** Analytical conditions and results of the fourteen SARS-CoV-2 libraries analysed in this study.

			Library Preparation		Library Size Assessment and Quantification				MPS Coverage Analysis				
Chip #	Sample Type	Sample Name #	RT Protocol	Number of PCR Cycles	Range (bp)	Conc. [pg/µl]	Region Molarity [pmol/l]	% of total	Mapped Reads	% On Target	Mean Depth	% Uniformity	AccessionNumber *
1	isolate	73696	A	12	200–370	470	2730	36.32	5173083	99.99	34,209	96.70	MT483875
1	isolate	77488	A	12	200–370	678	4000	43.92	1822450	99.99	12,087	96.70	EPI_ISL_417491
1	isolate	73696	A	20	200–370	3150	19,400	63.55	15465	100.00	108.9	70.75	N/A
1	isolate	77488	A	20	200–370	4150	25,800	67.96	8038	100.00	57.1	54.86	N/A
2	isolate	71205	A	12	200–370	1360	7610	40.23	869002	99.99	5727	97.77	MT479216
2	isolate	73702	A	12	200–370	983	5350	31.8	2403510	99.99	15,647	97.67	MT483881
2	isolate	78955	A	12	200–370	1550	8580	40.22	1192010	99.99	7757	97.36	MT483880
2	isolate	78952	A	12	200–370	1210	6600	45.75	1257760	99.99	8185	96.60	MT483867
2	isolate	32898	A	12	200–370	937	5300	35.19	2173810	99.99	14,257	98.02	MT483878
3	isolate	34117	A	12	200–370	1050	5900	36.44	1255377	100.00	8376	97.08	MT483877
3	isolate	34104	A	12	200–370	886	4860	45.06	2371944	99.99	15,651	97.91	MT483876
3	isolate	43950	A	12	200–370	1240	7610	52.75	1317649	99.99	8689	98.12	MT483879
-	nasopharyngeal swab	78955-sw1	A	12	200–370	102	657	5.5	-	-	-	-	N/A
3	nasopharyngeal swab	78955-sw	B	12	200–370	223	1190	46.47	2659704	97.98	16944	96.98	MT483874
-	nasopharyngeal swab	78955-sw0.1	B	12	200–370	12	71.2	6.73	-	-	-	-	N/A
-	nasopharyngeal swab	78955-sw0.1	B	20	200–370	78.6	467	23.26	-	-	-	-	N/A
-	nasopharyngeal swab	78955-sw2	B	12	200–370	120	684	33.11	-	-	-	-	N/A

* Accession numbers refer to nucleotide sequences deposited in GenBank except for the 77,488 isolate whose sequence was deposited in Global Initiative on Sharing All Influenza Data (GISAID); N/A means not assigned due to bad sequencing data or non-sequenced sample; MPS: massively parallel sequencing.

**Table 2:** Run and alignment summary results of the three chips.

	1^st^ chip (4 libraries)			2^nd^ chip (5 libraries)			3^rd^ chip (4 libraries)		
**Addressable Wells**	**12,530,194**			**12,530,194**			**12,530,194**		
With ISPs	11,656,645	93.0%		12,016,993	95.9%		11,576,776	92.4%	
Live	11,656,144	100.0%		12,016,587	100.0%		11,574,388	100.0%	
Test Fragment	198,238	1.7%		125,540	1.0%		166,846	1.4%	
Library	11,457,906	98.3%		11,891,047	99.0%		11,407,542	98.6%	
**Library ISPs**	**11,457,906**			**11,891,047**			**11,407,542**		
Filtered: Polyclonal	3,503,557	30.6%		3,459,838	29.1%		2,932,238	25.7%	
Filtered: Low Quality	779,648	6.8%		424,333	3.6%		577,350	5.1%	
Filtered: Adapter Dimer	4,127	0.0%		2,610	0.0%		6,596	0.1%	
Final Library ISPs	**7,170,574**	**62.6%**		**8,004,266**	**67.3%**		**7,891,358**	**69.2%**	
**Read length**									
Mean	206 bp			201 bp			201 bp		
Median	219 bp			217 bp			217 bp		
Mode	223 bp			223 bp			223 bp		
**Alignment Summary**									
Total Reads	7,022,030			7,899,198			7,687,875	–	
Aligned Reads	7,019,036	100.0%		7,896,092	100.0%		7,604,674	98.9%	
Unaligned Reads	2,994	0.0%		3,106	0.0%		83,201	1.1%	
**Alignment Quality**	**AQ17**	**AQ20**	**Perfect**	**AQ17**	**AQ20**	**Perfect**	**AQ17**	**AQ20**	**Perfect**
Total Number of Bases (Mbp)	1.37 G	1.3 G	1.06 G	1.53 G	1.49 G	1.25 G	1.47 G	1.43 G	1.21 G
Mean Length (bp)	197	191	160	196	192	167	197	194	169
Longest Alignment (bp)	368	366	358	367	366	358	362	362	358
Mean Coverage Depth	20.3	19.4	15.7	22.8	22.1	18.7	21.9	21.3	18.0

ISP: Ion Sphere Particles.
